# Trends in Diabetes and Cardiometabolic Conditions in a Canadian First Nation Community, 2002–2003 to 2011–2012

**DOI:** 10.5888/pcd11.140334

**Published:** 2014-11-13

**Authors:** Natalie D. Riediger, Lisa M. Lix, Virginia Lukianchuk, Sharon Bruce

**Affiliations:** Author Affiliations: Natalie D. Riediger, Department of Community Health Sciences and Manitoba First Nations Centre for Aboriginal Health Research, University of Manitoba, Winnipeg, Manitoba, Canada; Lisa M. Lix, Department of Community Health Sciences, University of Manitoba, Winnipeg, Manitoba, Canada; Virginia Lukianchuk, Sandy Bay Health Centre, Sandy Bay Ojibway First Nation, Manitoba, Canada.

## Abstract

**Introduction:**

The burden of diabetes and cardiovascular disease among the Canadian First Nation population is disproportionately high compared with the general Canadian population. Continuous monitoring of the diabetes epidemic among the Canadian First Nations population is necessary to inform public health practice. The purpose of the study was to compare the prevalence of diabetes and cardiometabolic conditions in a Manitoba First Nation between 2 periods.

**Methods:**

Study data were from 2 diabetes screening studies in Sandy Bay Ojibway First Nation in Manitoba, collected in 2002–2003 and 2011–2012. All adults aged 18 years or older were invited to participate in both studies. Crude and sex- and age-standardized prevalence of diabetes and cardiometabolic conditions for each period were estimated and compared with each other by using χ^2^ tests.

**Results:**

Sex- and age-standardized prevalence of diabetes was estimated at 39.4% (95% confidence interval [CI], 35.1–43.8) in 2002–2003 and was not significantly different (*P = .*99) in 2011–2012. Sex- and age-standardized obesity prevalence was significantly lower in 2011–2012, at 48.7% (95% CI, 44.6–52.7), compared with 60.8% (95% CI, 56.4–65.2) in 2002–2003 (*P* < .001). However, this finding was accounted for by a lower prevalence of obesity among men aged 40 to 49 and aged 50 years or older in 2011–2012 compared with 2002–2003. Sex- and age-standardized prevalence of hypertension (*P = .*97), abdominal obesity (*P = .*26), dyslipidemia (*P = .*73), and metabolic syndrome (*P = .*67) were not significantly different between periods. Significantly higher crude prevalence of obesity, abdominal obesity, dyslipidemia, and metabolic syndrome among women compared with men persisted from 2002–2003 to 2011–2012.

**Conclusion:**

The diabetes epidemic remains a serious problem in this First Nation community. The gap in cardiometabolic burden between men and women has also persisted.

## Introduction

The prevalence of diabetes and cardiovascular disease among the Canadian First Nation population is disproportionately high compared with the general Canadian population, and the prevalence has increased in recent years (1–3). Much research on the diabetes epidemic used administrative data, which are useful for estimating the burden. However, administrative data are derived from cases of physician-diagnosed diabetes, which is problematic given the known significant number of undiagnosed cases. Administrative data also cannot provide information on comorbid conditions such as dyslipidemia, obesity, abdominal obesity, and metabolic syndrome (4,5)

Another feature of the diabetes epidemic is the geographic heterogeneity of burden among First Nations people. Among Canadian First Nations people living on-reserve, the age-standardized prevalence of diabetes was 17.2% in 2008–2009 (6). In Saskatchewan, the age-standardized prevalence of diabetes among First Nations people was 20.3% for women and 16.0% for men in 2005 (1). Among urban and rural First Nations from Alberta, the sex- and age-standardized prevalence of diabetes in 2006 was 11.5% and 14.7%, respectively (3). In Manitoba, the age-standardized prevalence in 1998 among First Nations people was 24.9% among women and 17.0% among men (2). Given the available data, both administrative and community-based (7), Manitoba First Nations people have a high burden of diabetes compared with First Nations people of other provinces. In addition, rates of diabetes vary significantly by tribal council in Manitoba. The Dakota Ojibway Tribal Council, of which the study community is a member, has the highest age- and sex-standardized prevalence of physician-diagnosed diabetes in Manitoba, at 24.9% from 1996–1997 through 1998–1999 (4). 

A study completed in 2002–2003 in the Sandy Bay First Nation indicated that the crude prevalence of diabetes was nearly 30% of the adult population (5), with an age-standardized rate likely to be substantially higher. Therefore, the study community is a population with a high burden of disease that requires continued monitoring. The purpose of this study is to describe the burden of diabetes and other cardiometabolic conditions in a Manitoba First Nation community and to describe how the burden has changed from 2002–2003 to 2011–2012.

## Methods

### Setting

The study community is Sandy Bay Ojibway First Nation, located in southwest Manitoba, approximately 200 kilometers northwest of Winnipeg. This community is accessible year round by road. The total on-reserve population in 2011 was approximately 4,100 people, 50% of whom are younger than 19 years.

### Design

Data from the 2002–2003 and 2011–2012 diabetes screening studies were included in the repeated cross-sectional design. Details about the 2002–2003 screening study can be found elsewhere (5). Briefly, Sandy Bay First Nation invited researchers to conduct a diabetes screening study. Fasting blood samples were taken, and anthropometric and questionnaire data were collected. Data collection occurred between October 2002 and December 2003. The second cross-sectional study, conducted between July 2011 and June 2012, also took fasting blood samples and collected anthropometric and questionnaire data. Both studies were approved by the University of Manitoba Health Research Ethics Board.

The study used a community-based participatory framework (8). The community identified the problem and sought out university researchers. A diabetes advisory group, including members from the Health Centre, community members, and university researchers, has overseen all aspects of the studies since 2002. The community uses the results when attending meetings with government to provide evidence for action.

### Sampling

All adults who were aged 18 years or older and not pregnant were invited to participate in both study periods (ie, study sample was a convenience sample). Participants had to be registered members of Sandy Bay Ojibway First Nation or a registered member of another First Nation but living in Sandy Bay. Recruitment was conducted through advertisement at the Community Health Centre and via a local radio station, word of mouth, and home visits from community research assistants. Transportation was offered to all participants.

### Outcomes

Venous blood samples were drawn by a registered nurse after a minimum 12-hour fast. Methods for measurement of glucose, hemoglobin A1c (HbA1c), triglycerides, and high-density lipoprotein (HDL) cholesterol have been described previously (5). Blood pressure was assessed by trained research assistants using an automated blood pressure monitor (Omron Corporation). At least 2 blood pressure readings were taken and averaged. Anthropometric measures — height, weight, and waist circumference — were taken using standard techniques (9).

Diabetes was defined by self-report, currently taking an oral hypoglycemic medication, or having a fasting blood glucose of 7.0 mmol/L or higher (10,11). Impaired fasting glucose (IFG) was defined as a fasting blood glucose between 6.1 and 6.9 mmol/L (12). Obesity was defined as a body mass index (BMI, measured as weight in kilograms divided by the square of height in meters) of 30 kg/m^2^ or higher (13). Hypertension was defined as a previous diagnosis of hypertension or a systolic blood pressure (SBP) higher than 140 mm Hg or diastolic blood pressure (DBP) higher than 90 mm Hg; for participants with diabetes, hypertension was defined as an SBP of 130 mm Hg or more or a DBP of 80 mm Hg or more (10,11). Dyslipidemia was defined as a fasting plasma triglyceride of 1.7 mmol/L or higher and a fasting plasma HDL of less than 1.03 mmol/L (for men) or a plasma HDL of less than 1.30 mmol/L (for women) (14). Metabolic syndrome was defined as meeting 3 or more of the following criteria: waist circumference of 102 cm or more for men and of 88 cm or more for women, a fasting blood glucose of 5.6 mmol/L or more (or previous diabetes diagnosis), a fasting triglyceride level of 1.7 mmol/L or more, an HDL cholesterol level of less than 1.03 mmol/L for men or less than 1.30 mmol/L for women, and a blood pressure of 130/85 mm Hg or more or a previous diagnosis of hypertension (14).

### Statistical analysis

Sociodemographic characteristics, such as age, sex, highest level of education, and employment (either full-time or part-time), are reported for each of the samples using frequencies, means, and standard deviations (SDs). Differences in mean age between periods were tested using an independent sample *t* test. Differences in proportions of other characteristics between periods were tested using χ^2^ test.

Sex- and age-stratified crude prevalence of diabetes and cardiometabolic conditions were estimated for each period. Sex- and age-standardized prevalence were also estimated for each cardiometabolic condition, using the 2010 Canadian population estimates, participants being aged 18 years or older (15), and the direct method of standardization. Age groups were 18 to 29, 30 to 39, 40 to 49, and 50 years or older (5). To determine differences in diabetes and cardiometabolic conditions between the 2 periods, nonlinear mixed-model with random intercept were used to account for dependency in the data. However, mixed models did not converge because of the limited number of participants with repeated measures (n = 171). As a result, differences in sex- and age-standardized prevalence of each cardiometabolic condition between periods were determined using χ^2^ tests. Sex differences in crude prevalence of each respective cardiometabolic condition for each period were also assessed using χ^2^ test. Differences in prevalence between periods were further explored to determine differences in odds of a condition by using logistic regression adjusting for age group, sex, and other relevant conditions. Analyses were conducted for separate age groups when appropriate.

To further explore differences over time in the burden of diabetes, we also tested for differences in fasting blood glucose, HbA1c, and age at diagnosis between periods. Generalized linear models with gamma distribution to account for a skewed distribution were used. Analyses for age at diagnosis were conducted only for those with diabetes. Control variables were age group, sex, and presence of diabetes (for fasting blood glucose and HbA1c). Interaction effects were also explored, and stratified analyses were conducted to understand changes in health status between the periods.

All statistical analyses were conducted using SPSS version 22 (IBM Corporation). All tests of significance were conducted using an α level of .05.

## Results

A total of 482 community members (44% of the eligible population) participated in 2002–2003. The sample was representative of the community according to age, sex, and employment status (5). The 2011–2012 sample totaled 596 participants, or 28% of the eligible population (Table 1). The percentage of the eligible population was equal for men and women (27.8% for men and 27.3% for women). The 2011–2012 sample was representative of the population according to age group and sex. The mean age of the 2011–2012 sample was significantly younger than the mean age of the 2002–2003 sample (*P = .*007); both samples had a similar proportion of men and women (*P = .*13). The 2011–2012 sample was more highly educated than the 2002–2003 sample, but the 2011–2012 sample had a significantly lower employment rate than the 2002–2003 sample (Table 1).

**Table 1:** Chracteristics of Participants in a Cross-Sectional Study of Diabetes and Cardiometabolic Conditions, Sandy Bay First Nation Residents^a^, 2002–2003 and 2011–2012

Characteristic	2002–2003 (n = 482)	2011–2012 (n = 596)	*P* Value^b^
**Mean age, y (SD)**	37.8 (12.3)	35.7 (12.9)	.007
**Age group, y**			
18–29	142 (29.5)	237 (39.8)	.001
30–39	144 (29.9)	127 (21.3)	
40–49	108 (22.4)	134 (22.5)	
≥50	88 (18.3)	98 (16.4)	
**Sex**			
Male	230 (47.7)	312 (52.3)	.13
Female	252 (52.3)	284 (47.7)	
**Education leve^c^**			
<grade 9	248 (53.0)	159 (27.2)	<.001
≥grade 9	220 (47.0)	426 (72.8)	
**Employed**			
Yes	137 (28.8)	123 (20.6)	.002
No	338 (71.2)	473 (79.4)	

Abbreviation: SD, standard deviation.

a Data presented as no. (%), unless otherwise indicated.

b *P* value for mean age calculated using independent sample *t* test for difference between cross-sectional samples; all other *P* values calculated using χ^2^ test of independence cross-sectional samples.

c Based on median split in 2003 sample.

Sex- and age-specific prevalence of diabetes and other cardiometabolic conditions are reported in Table 2. The crude prevalence of diabetes was 29.0% (95% confidence interval [CI], 25.0%–33.1%) in 2002–2003 and 25.9% (95% CI, 22.4%–29.4%) in 2011–2012. An additional 6.2% (95% CI, 4.1%–8.4%) had IFG in 2002–2003 and 6.1% (95% CI, 4.2%–8.0%) in 2011–2012. Sex- and age-standardized prevalence of diabetes was 39.4% (95% CI, 35.1%–43.8%) in 2002–2003 and 39.2% (95% CI, 35.3%–43.1%) in 2011–2012 (Figure); the change between periods was not significant (*P = .*99). During both study periods, the crude diabetes prevalence was higher among women than men, but the difference was not significant. In 2002–2003, 7.3% (95% CI, 5.0%–9.6%) of the sample had undiagnosed diabetes; in 2011–2012, 6.1% (95% CI, 4.2%–8.0%) of the total sample had undiagnosed diabetes. Of those with diabetes, 25.4% (95% CI, 18.1%–32.7%) met the HbA1c target of <7.0% (10) in 2002–2003, and 26.0% (95% CI, 19.1%–32.9%) met the target in 2011–2012.

**Table 2:** Crude Sex- and Age-Specific Prevalence of Cardiometabolic Conditions Among Sandy Bay First Nation Residents, 2002–2003 and 2011–2012

Disease/Condition^a^	Age group, y	Men, % (95% CI)		Women, % (95% CI)	
		2002–2003	2011–2012	2002–2003	2011–2012
Diabetes (n = 1,077)	All ages	27.0 (21.2–32.7)	24.8 (20.0–29.6)	31.0 (25.2–36.7)	27.1 (21.9–32.3)
	18–29	8.2 (1.9–14.5)	5.2 (1.4–8.9)	15.9 (7.3–24.6)	9.8 (4.0–15.6)
	30–39	23.1 (12.8–33.3)	25.4 (14.3–36.5)	20.3 (11.4–29.1)	23.9 (13.7–34.1)
	40–49	36.7 (23.2–50.2)	33.8 (22.6–45.1)	33.9 (21.8–46.0)	31.8 (20.6–43.1)
	**≥**50	53.5 (38.6–68.4)	65.3 (52.0–78.6)	68.9 (55.4–82.4)	61.2 (47.6–74.9)
Hypertension (n = 1,063)	All ages	37.3 (31.0–43.6)	44.0 (38.5–49.5)	44.0 (37.8–50.3)	34.3 (28.7–39.8)
	18–29	9.6 (2.8–16.3)	22.4 (15.3–29.4)	26.5 (16.0–37.0)	14.9 (7.9–21.8)
	30–39	38.5 (26.6–50.3)	50.0 (37.3–62.7)	32.1 (21.7–42.4)	31.3 (20.2–42.5)
	40–49	47.9 (33.8–62.0)	51.5 (39.5–63.6)	54.5 (41.4–67.7)	39.4 (27.6–51.2)
	**≥**50	71.4 (57.8–85.1)	85.7 (75.9–95.5)	81.0 (69.1–92.8)	71.4 (58.8–84.1)
Obesity (n = 1,061)	All ages	47.6 (41.1–54.1)	35.6 (30.3–40.9)	65.1 (59.1–71.2)	60.2 (54.5–65.9)
	18–29	35.6 (24.6–46.6)	36.6 (28.4–44.7)	61.8 (50.2–73.3)	51.0 (41.3–60.7)
	30–39	43.1 (31.0–55.1)	41.7 (29.2–54.1)	61.5 (50.7–72.3)	67.2 (55.9–78.4)
	40–49	62.5 (48.8–76.2)	27.3 (16.5–38.0)	64.8 (52.1–77.6)	66.7 (55.3–78.0)
	**≥**50	58.5 (43.5–73.6)	36.7 (23.2–50.2)	78.0 (65.4–90.7)	61.2 (47.6–74.9)
Dyslipidemia (n = 1,077)^a^	All ages	25.2 (19.6–30.8)	20.3 (15.8–24.7)	36.5 (30.6–42.5)	31.0 (25.6–36.4)
	18–29	16.4 (7.9–24.9)	15.6 (9.4–21.7)	27.5 (17.0–38.1)	18.6 (11.1–26.2)
	30–39	29.2 (18.2–40.3)	33.9 (21.8–46.0)	35.4 (24.9–46.0)	34.3 (23.0–45.7)
	40–49	36.7 (23.2–50.2)	17.6 (8.6–26.7)	39.0 (26.5–51.4)	28.8 (17.9–39.7)
	**≥**50	20.9 (8.8–33.1)	20.4 (9.1–31.7)	48.9 (34.3–63.5)	55.1 (41.2–69.0)
Metabolic syndrome (n = 1,050)	All ages	47.3 (40.8–53.9)	43.5 (37.9–49.0)	61.0 (54.8–67.2)	56.0 (50.2–61.8)
	18–29	28.8 (18.4–39.2)	29.5 (21.8–37.3)	45.5 (33.4–57.5)	34.0 (24.7–43.3)
	30–39	40.0 (28.1–51.9)	54.2 (41.5–66.9)	55.1 (44.1–66.2)	58.2 (46.4–70.0)
	40–49	68.1 (54.8–81.4)	43.9 (32.0–55.9)	69.2 (56.7–81.8)	63.6 (52.0–75.2)
	**≥**50	68.3 (54.0–82.5)	67.3 (54.2–80.5)	87.5 (77.3–97.7)	87.8 (78.6–96.9)

Abbreviation: CI, confidence interval.

a Represents the sample, for both periods combined, for which data were available for each disease/condition. This value includes repeated measures.

**Figure:**
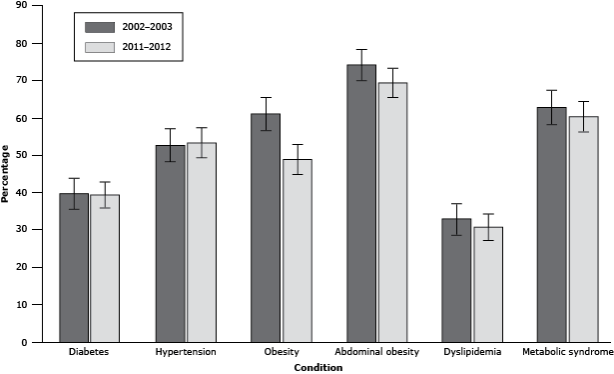
Sex- and age-standardized prevalence of diabetes and cardiometabolic conditions in Sandy Bay First Nation population, 2002–2003 and 2011–2012. **Table:** 

Condition	Prevalence (95% Confidence Interval)	
	2002–2003	2011–2012
Diabetes	39.4 (35.1–43.8)	39.2 (35.3–43.1)
Hypertension	52.4 (47.9–56.9)	53.1 (49.1–57.2)
Obesity	60.8 (56.4–65.2)	48.6 (44.6–52.7)
Abdominal obesity	73.8 (69.8–77.8)	69.1 (65.3–72.8)
Dyslipidemia	32.6 (28.5–36.8)	30.4 (26.8–34.1)
Metabolic syndrome	62.5 (58.1–66.9)	59.9 (56.0–63.9)

Among participants with diabetes, including those with newly diagnosed diabetes, there was not a significant difference in age at diagnosis between periods (*P = .*15), independent of age group and sex. In 2002–2003, the mean age at diagnosis was 38.6 years (SD, 11.6 y) for men and 38.0 years (SD, 12.7 y) for women; in 2011–2012, the mean ages were 37.9 years (SD, 10.9 y) for men and 37.0 years (SD, 11.2 y) for women. Fasting blood glucose was significantly higher (*P < .*001) among participants without diabetes in 2011–2012, at 5.39 mmol/L (SD, 0.49 mmol/L), compared with 5.21 mmol/L (SD, 0.58 mmol/L) in 2002–2003, independent of age group or sex. HbA1c was significantly higher (*P* < .001) among participants without diabetes in 2011–2012 (5.78% [SD, 0.34%]) compared with 2002–2003 (5.60% [SD, 0.42%]), independent of age group or sex.

The crude prevalence of hypertension in 2002–2003 was 40.8% and 39.4% in 2011–2012. Crude prevalence was not significantly different between men and women in 2002–2003 (*P = .*14). However, the crude prevalence of hypertension was significantly higher among men than women in 2011–2012 (*P = .*015). The sex- and age-standardized prevalence of hypertension was 52.4% in 2002–2003 and 53.1% in 2011–2012 (Figure); the prevalence between periods was not significantly different (*P = .*97). Undiagnosed hypertension was found in 13.6% of the sample in 2002–2003 and in 18.6% in 2011–2012. Among participants with diabetes, 82.2% had hypertension and 31.0% had undiagnosed hypertension in 2002–2003, and 78.0% had hypertension and 34.4% had undiagnosed hypertension in 2011–2012.

The crude prevalence of obesity was 56.6% in 2002–2003 and 47.4% in 2011–2012. During both periods, the sex-specific crude prevalence was significantly higher among women than men (*P < .*001 for both). The sex- and age-standardized prevalence was 60.8% in 2002–2003 and 48.6% in 2011–2012 (Figure). The sex- and age-standardized prevalence of obesity was significantly lower in 2011–2012 (*P < .*001). Differences in crude prevalence between periods varied according to age and sex (Table 2). For example, among men aged 40 to 49 years, crude prevalence of obesity was 62.5% in 2002–2003 and 27.3% in 2011–2012. Logistic regression models confirmed a significantly lower odds of obesity in 2011–2012 compared with 2002–2003 among men aged 40–49 and ≥50 years (data not shown). The models for men were further adjusted by diabetes status, because diabetes can result in weight loss, as well as hypertension. However, these adjustments did not change the significant difference in odds of obesity between time periods among men in either age group.

The crude prevalence of abdominal obesity was 67.4% in 2002–2003 and 64.6% in 2011–2012. The sex- and age-standardized prevalence was 73.8% in 2002–2003 and 69.1% in 2011–2012 (Figure), which was not significantly different (*P = .*26). Sex-specific crude prevalence was 53.1% for men and 81.0% for women in 2002–2003 and 47.6% for men and 83.0% for women in 2011–2012. Again, prevalence was significantly higher among women than men in each period (*P < .*001 for both).

The crude prevalence of dyslipidemia in 2002–2003 was 31.1% and 25.4% in 2011–2012. The sex-specific prevalence was significantly higher among women than men in each period (2002–2003, *P = .*007; 2011–2012, *P = .*003). The sex- and age-standardized prevalence was 32.6% in 2002–2003 and 30.4% in 2011–2012 (Figure) and not significantly different (*P = .*73).

The crude prevalence of metabolic syndrome in 2002–2003 was 54.3% and 49.5% in 2011–2012. The sex-specific prevalence was higher for women compared with men in each period (2002–2003, *P = .*003; 2011–2012, *P = .*002). The sex- and age-standardized prevalence of metabolic syndrome was 62.5% in 2002–2003 and 59.9% in 2011–2012 (Figure) and not significantly different (*P = .*67).

## Discussion

Overall, few substantial changes at the population level were found for cardiometabolic burden from 2002–2003 to 2011–2012 in this Canadian First Nation community. The sex- and age-standardized diabetes prevalence was not significantly different between periods. It is difficult to make statements about the trajectory of the diabetes epidemic on the basis of only 2 points. However, the absolute burden of disease, with a sex- and age-standardized diabetes prevalence at 39.2% in 2011–2012, is considerable. Another feature of the epidemic in this community was the adverse changes in fasting glucose among those without diabetes. This finding is concerning because it may indicate a decrease in health status among those without diabetes, many of whom are young. An additional note on the diabetes epidemic in Sandy Bay is the persistence of a high rate of undiagnosed diabetes and hypertension and young age at diagnosis. Diagnosis of diabetes is critical in managing blood glucose and in preventing and delaying complications (16), particularly among young people when risk of complications is greater (17).

The implications of significant differences in sociodemographic factors between the 2 study periods are difficult to interpret. Crude rates of diabetes and most conditions were lower in 2011–2012 because of the younger age of the study population relative to 2002–2003. However sex- and age-standardized rates were similar. Although educational levels increased, translation into greater employment has not occurred because of limited opportunities in the community and surrounding area. Therefore, social conditions likely remain a contributor to cardiometabolic outcomes in the study community.

Sex-specific patterns are another feature of the epidemic. The literature has consistently reported higher rates of diabetes among First Nations women than among First Nations men (1–3). In contrast to the findings published in the literature (1–3), we found that the prevalence of diabetes was not significantly different between men and women in either period. This may be a feature of a more advanced epidemic, given the reported reduction in the diabetes gap between men and women over time in Saskatchewan (1). Similarly, an increasing incidence of diabetes over time for First Nations men compared with a plateau for First Nations women in Manitoba during the 1990s was reported (2). The higher prevalence of other cardiometabolic conditions found among women than among men in this study may partially explain the higher risk associated with diabetes on cardiovascular outcomes for women reported in other populations (18). This explanation is also consistent with previous reports suggesting that women experience more pronounced adverse changes in lipid profile in response to diabetes compared with men (19,20). This sex difference may also partially explain the larger gap in rates of cardiovascular mortality observed between Canadian First Nations women and non-First Nations women compared with their male counterparts (21).

Although the lower prevalence of obesity in 2011–2012 compared with 2002–2003 was encouraging, this finding was mostly accounted for by the lower prevalence among middle-aged men. This difference over time among middle-aged men was not accounted for by diabetes or hypertension. The lower prevalence of obesity among men aged 40 to 49 also coincided with a lower prevalence of dyslipidemia and metabolic syndrome, which is not surprising given their known associations. However, prevalence of diabetes and hypertension were not lower in 2011–2012 in this age group; therefore, this apparent population improvement should be interpreted with caution.

There are several strengths and limitations of this study. First, it was conducted using a community-based participatory framework. The researchers will continue to work with the community to translate the findings and support policy changes at the community level. Second, this study provides a rich description of changes, or lack thereof, of an important public health problem in this population, which cannot be ascertained using administrative data. Sample size, issues of sample dependence, and multiple comparisons may be considered limitations.

Primary care services need to be strengthened and additional public health efforts are needed to address the diabetes and cardiometabolic burden in this community. The burden of disease is troublesome, given the early age at diagnosis of diabetes and diabetes-related conditions. Of particular concern is the higher prevalence of several of these conditions among women in the community. This study provides a benchmark for the community to use when planning, implementing, and evaluating future interventions.

## References

[1] Dyck R , Osgood N , Lin TH , Gao A , Stang MR . Epidemiology of diabetes mellitus among First Nations and non-First Nations adults. CMAJ 2010;182(3):249–56. 10.1503/cmaj.090846 20083562 PMC2826466

[2] Green C , Blanchard JF , Young TK , Griffith J . The epidemiology of diabetes in the Manitoba-Registered First Nation population: current patterns and comparative trends. Diabetes Care 2003;26(7):1993–8. 10.2337/diacare.26.7.1993 12832301

[3] Johnson JA , Vermeulen SU , Toth EL , Hemmelgarn BR , Ralph-Campbell K , Hugel G , Increasing incidence and prevalence of diabetes among the Status Aboriginal population in urban and rural Alberta, 1995–2006. Can J Public Health 2009;100(3):231–6. 19507729 10.1007/BF03405547 PMC6973863

[4] Martens PJ , Martin BD , O'Neil JD , MacKinnon M . Diabetes and adverse outcomes in a First Nations population: associations with healthcare access, and socioeconomic and geographic factors. Can J Diab 2007;31(3):223–32. 10.1016/S1499-2671(07)13009-4

[5] Bruce SG , Young TK . Prevalence and risk factors for neuropathy in a Canadian First Nation community. Diabetes Care 2008;31(9):1837–41. 10.2337/dc08-0278 18509208 PMC2518355

[6] Pelletier C , Dai S , Roberts KC , Bienek A , Onysko J , Pelletier L . Report summary: diabetes in Canada: facts and figures from a public health perspective. Chronic Dis Inj Can 2012;33(1):53–4. 23294922

[7] Horn OK , Jacobs-Whyte H , Ing A , Bruegl A , Paradis G , Macaulay AC . Incidence and prevalence of type 2 diabetes in the First Nation Community of Kahnawáke, Quebec, Canada, 1986-2003. Can J Public Health 2007;98(6):438–43. 19039878 10.1007/BF03405434 PMC6975571

[8] Minkler M , Wallerstein N , editors. Community-based participatory research for health: from process to outcomes. 2nd edition. San Francisco (CA): John Wiley and Sons; 2008.

[9] Canadian Society for Exercise Physiology. The Canadian physical activity, fitness and lifestyle appraisal. Ottawa (ON): Canadian Society for Exercise Physiology; 1996.

[10] American Diabetes Association. Executive summary: standards of medical care in diabetes — 2013. Diabetes Care 2013;36(Suppl 1):S4–10. 10.2337/dc13-S004 23264424 PMC3537272

[11] Clinical Guidelines Task Force Global Guideline for Type 2 Diabetes. Brussels (BE): International Diabetes Federation; 2012. http://www.idf.org/sites/default/files/IDF-Guideline-for-Type-2-Diabetes.pdf

[12] Definition and diagnosis of diabetes mellitus and intermediate hyperglycemia. Report of a WHO/IDF consultation. Geneva (CH): World Health Organization, International Diabetes Federation; 2006.

[13] Obesity and overweight. Fact sheet number 311; Geneva (CH): World Health Organization; 2014. http://www.who.int/mediacentre/factsheets/fs311/en/. Accessed October 4, 2013.

[14] Alberti KG , Eckel RH , Grundy SM , Zimmet PZ , Cleeman JI , Donato KA , Harmonizing the metabolic syndrome. A joint interim statement of the International Diabetes Federation Task Force on Epidemiology and Prevention; National Heart, Lung, and Blood Institute; American Heart Association; World Heart Federation; International Atherosclerosis Society; and International Association for the Study of Obesity. Circulation 2009;120(16):1640–5. 10.1161/CIRCULATIONAHA.109.192644 19805654

[15] Annual demographic estimates: Canada, provinces and territories. Ottawa (ON): Statistics Canada; 2011. http://www.statcan.gc.ca/pub/91-209-x/2011001/article/11511/figures/desc/desc01-eng.htm.

[16] Young TK , Mustard C . Undiagnosed diabetes: does it matter? CMAJ 2001;164(1):24–8. 11202663 PMC80628

[17] Dart AB , Martens PJ , Rigatto C , Brownell MD , Dean HJ , Sellers EA . Earlier onset of complications in youth with type 2 diabetes. Diabetes Care 2014;37(2):436–43. 10.2337/dc13-0954 24130346

[18] Natarajan S , Liao Y , Cao G , Lipsitz SR , McGee DL . Sex differences in risk for coronary heart disease mortality associated with diabetes and established coronary heart disease. Arch Intern Med 2003;163(14):1735–40. 10.1001/archinte.163.14.1735 12885690

[19] Howard BV , Cowan LD , Go O , Welty TK , Robbins DC , Lee ET . Adverse effects of diabetes on multiple cardiovascular disease risk factors in women: the Strong Heart Study. Diabetes Care 1998;21(8):1258–65. 10.2337/diacare.21.8.1258 9702430

[20] Bittner V . Lipoprotein abnormalities related to women’s health. Am J Cardiol 2002;90(8 Suppl):77–84. 10.1016/S0002-9149(02)02637-1 12419484

[21] Tjepkema M , Wilkens R , Goedhuis N , Pennock J . Cardiovascular disease mortality among First Nations people in Canada, 1991–2001. Chronic Dis Inj Can 2012;32(4):200–7. 23046802

